# Body mass index and the risk of low femoral artery puncture in coronary angiography under fluoroscopy guidance

**DOI:** 10.1097/MD.0000000000010070

**Published:** 2018-03-02

**Authors:** Minsuk Kim, Myung-A Kim, Hack-Lyoung Kim, Won-Jae Lee, Woo-Hyun Lim, Jae-Bin Seo, Sang-Hyun Kim, Joo-Hee Zo

**Affiliations:** a Division of Cardiology, Department of Internal Medicine, Seoul National University Bundang Hospital, Seongnam; b Seoul National University College of Medicine; c Division of Cardiology, Department of Internal Medicine, Boramae Medical Center, Seoul, Korea.

**Keywords:** body mass index, coronary angiography, femoral artery, fluoroscopy, puncture

## Abstract

The inferior border of the femoral head (IBFH) is widely used as a landmark in femoral artery puncture during invasive coronary angiography (ICA). However, application of this technique can be challenging especially in obese patients. This study was performed to investigate the association between body mass index (BMI) and the risk of low puncture in femoral artery puncture.

A total of 464 patients (64.8 ± 11.1 years, 55.8% male) who underwent ICA via trans-femoral access were retrospectively reviewed. IBFH was used as a landmark for a skin nick and the femoral artery cannulation site was confirmed by femoral angiography. Cannulation at the bifurcation of the common femoral artery (CFA) or below were considered low puncture.

Twenty-nine patients (5.8%) were identified as having an angiographically high CFA bifurcation and low femoral artery puncture occurred in 27 (93.1%) patients of them. Among patients with normal bifurcation (n = 464), low puncture occurred in 74 (15.9%) patients. Underweight (BMI < 18.5 kg/m^2^) or obese (BMI ≥ 30 kg/m^2^) patients were more common in the low puncture group than in the proper puncture group (36.5% vs. 5.9%, *P* < .001). Multivariable analysis showed underweight or obesity (odd ratio, 9.10; 95% confidential interval, 4.77–17.35; *P* < .001) was an independent risk factor of low puncture even after controlling for clinical covariates. The average distance from IBFH to the CFA puncture site was shorter in patients with underweight (1.74 ± 0.71 cm) or obesity (1.75 ± 0.60 cm) than in those with normal BMI or overweight (2.07 ± 0.83 cm) (*P* = .030). Trigonometric calculation showed that the average distance from IBFH to the CFA puncture site was 0.5 to 2.59 cm (mean = 1.32 cm) shorter in underweight patients compared with those of normal weight or overweight patients.

In patients with normal CFA bifurcation, underweight or obesity were associated with increased risk of low puncture. The puncture site should be chosen about 1 finger width more proximal to IBFH for ICA in such patients.

## Abstract 2

Supplemental Digital Content is available in the text

## 1 Introduction

Coronary artery disease (CAD) is the leading cause of death in many developed countries.^[1]^ Invasive coronary angiography (ICA) and percutaneous coronary intervention play a pivotal role in the diagnosis and treatment of CAD. The most commonly used vascular access for ICA is the radial and common femoral arteries (CFA). Although there has been a recent increase in the use of the radial approach with lower complication rates at the access site,^[2]^ the traditional CFA approach is still the main vascular access for ICA and interventional procedures.^[3]^ Compared with the radial approach, the CFA approach allows the use of a large-diameter catheter and a sheath, and is associated with a reduced volume of contrast agents, shorter procedural time, and less radiation exposure.^[2,4]^ However, it should be noticed that the risk of access site complications was significantly higher and more critical in patients with the femoral approach than in those with the radial approach.^[2,3,5]^ Therefore, it is important to find out how to reduce vascular access site complications, especially in the CFA approach.

An ideal femoral artery puncture site, between the CFA bifurcation and the points 1 to 2 cm below the inguinal ligament, has been suggested and widely used in the clinical field to minimize access site complications during CFA approach.^[6]^ High femoral artery puncture is associated with increased risk of retroperitoneal hemorrhage, and low femoral artery puncture is associated with increased risk of puncture site bleeding, pseudoaneurysm, arteriovenous fistula, and thrombosis.^[6,7]^ In addition, vascular closure devices cannot be applied to puncture sites that are not located in the CFA, which may interrupt early ambulation and decrease patient compliance.^[8]^ Therefore, choosing an ideal puncture point is very important. The most widely used puncture site identification method is fluoroscopic visualization using the inferior border of femoral head (IBFH) as the puncture entry site on the skin.^[6,9]^

As obesity is an independent risk factor for CAD,^[10,11]^ and obese patients tend to have higher chances to undergo ICA.^[12,13]^ However, obesity is associated with increased risk of vascular complications.^[13]^ In particular, the conventional femoral artery puncture technique under fluoroscopy-guidance seems to have higher risk of low puncture in obese patients than in the normal population.^[6,13]^ However, there have been lack of studies focusing whether body mass index (BMI) influence the risk of low puncture.

This study was performed to investigate the association between BMI and the risk of low puncture, and sought to find out an optimal puncture technique according to BMI in fluoroscopy-guided femoral artery puncture.

## 2 Materials and methods

### 2.1 Study population

This single-center study was performed at Boramae Medical Center (Seoul, Korea). Between January 2013 and August 2016, a total of 493 consecutive patients who underwent ICA and femoral angiography via the right CFA by a single cardiologist were identified, and their medical records were retrospectively reviewed. There were no exclusion criteria. The study protocol was reviewed and approved by the Institutional Review Board (IRB) of Boramae Medical Center. Written informed consent was waived by the IRB due to the retrospective study design and routine nature of information collected.

### 2.2 Procedures

**Figure 1:**
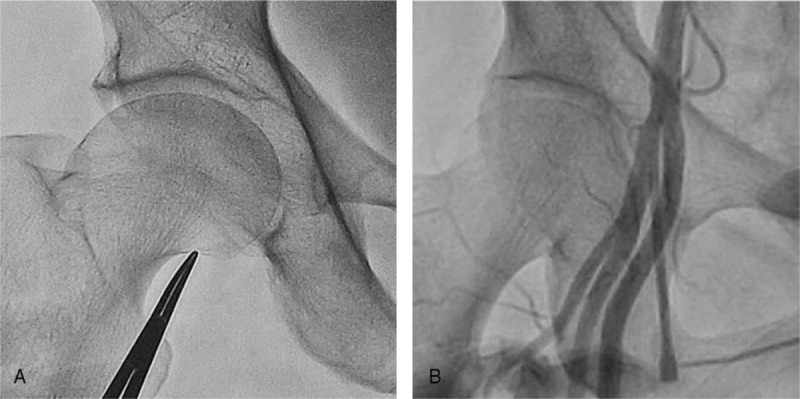
Femoral fluoroscopy indicating the inferior border of the femoral head (A) and femoral angiography of high bifurcation (B).

All CFA punctures were performed under fluoroscopy-guidance according to the conventional technique at the right CFA using a 6- or 7-Fr sheath.^[5,6]^ IBFH was aimed via fluoroscopy in the anteroposterior (AP) view, and a skin nick for femoral artery puncture was made at this point (Fig. 1A). A Seldinger needle (18 guage, 7 cm) was used to cannulate the femoral artery. Femoral artery angiography was obtained at the end of each ICA. To minimize image-related biases, biplane femoral angiography was performed with the femoral head at the center of the screen in the AP and/or oblique 60° projections. All angiographic measurements were made using INFINITT Cardiology PACS (Version 1.0.5.4 BN152, INFINITT Healthcare Co., Ltd, Seoul, South Korea). Femur head length (FHL) was defined as the distance between the most proximal point of the femoral head and IBFH in the AP view. Vertical distance from IBFH to the closest bifurcation point was measured on femoral angiography. “High bifurcation” was defined as femoral artery bifurcation originating within the proximal half of the femoral head (Fig. 1B).^[14]^ Puncture distance was measured vertically from IBFH to the midpoint of the femoral sheath at the puncture site on femoral angiography. The puncture site distal to any bifurcation of the CFA (superficial femoral artery, lateral circumflex femoral artery, and deep femoral artery) was considered low puncture. CFA size was measured perpendicular to the CFA on femoral angiography. We assumed a tortuous CFA as having at least 1 curvature more than 90° within the CFA on femoral angiography. Linear calcification on femoral angiography and hardness at the CFA puncture site was regarded as heavy calcification. Atheroma was detected as a halo-like ring of radiodensity in the CFA. Periprocedural complications were observed until discharge date. Skin discoloration at the puncture site was defined as bruise, and swelling of the skin with bruise was considered hematoma. Bleeding was defined as massive one at the puncture site that caused hemoglobin to drop more than 3 g/dL according to Bleeding Academic Research Consortium bleeding criteria.^[15]^ Pseudoaneurysm was diagnosed using imaging modalities, including ultrasound or computed tomography angiography. Blood transfusion was defined when more than 200 mL of packed red blood cells were administered.

### 2.3 Anthropometric data

BMI was calculated as the body weight (kg) divided by the square of the height (m^2^). Underweight was defined as under 18.5 kg/m^2^ and obesity was over 30.0 kg/m^2^ according to World Health Organization stratification.^[16]^ Body surface area was calculated by the following formula: (height [cm] × weight [kg]/3600)^1/2^.^[17]^

### 2.4 Statistical analysis

Variables are expressed as mean ± standard deviation or percentages. The mean values and proportions of variables between independent 2 groups were compared using Student *t* test and chi-squared test or Fisher exact test, respectively. Comparisons of mean values and proportions of variables among independent 3 groups were performed using 1-way analysis of variance and chi-squared test, respectively. Stepwise conditional logistic regression analysis was performed to determine independent risk factors for low puncture. The odds ratio (OR) with 95% confidence interval (CI) were evaluated to determine the relative risk (RR) of each suspected risk factor. A *P* value < .05 was considered statistically significant. Statistical analyses were performed using SPSS 18.0 (IBM Co, Armonk, NY).

## 3 Results

**Figure 2:**
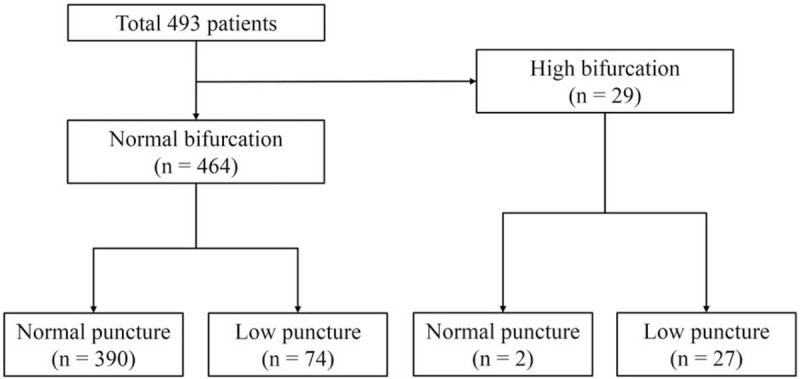
Classification of the study population.

Among 493 patients, 29 (5.8%) showed high bifurcation. These patients with high bifurcation were separated because conventional femoral artery puncture technique could not be expected to achieve successful cannulation. As expected, successful puncture was observed in only 2 patients (6.9%). Patients with normal bifurcation were divided into 2 groups: those with proper femoral artery puncture (proper puncture group, n = 390) and those with low femoral artery puncture (low puncture group, n = 74) (Fig. 2).

### 3.1 Comparisons between patients with normal and high bifurcation

Clinical and angiographic characteristics were compared between patients with normal and high bifurcation (Table 1). Females were more common in patients with high bifurcation compared to those with normal bifurcation (62.1% vs. 43.1%, *P* = .046), but age and anthropometric parameters showed no significant differences (*P* > .05 for each). BMI and body weight (underweight, normal or overweight, and obese) also showed no significant differences between patients with high and normal bifurcation. In patients with high bifurcation, FHL was shorter (5.59 ± 0.52 cm vs. 5.82 ± 0.59 cm, *P* = .032), and tortuous CFA were more common (17.2% vs. 3.2%, *P* < .001) compared to those with normal bifurcation. Patients with high bifurcation had a higher rate of low puncture compared with those with normal bifurcation (93.1% vs. 15.9%, *P* < .001).

**Table 1:**
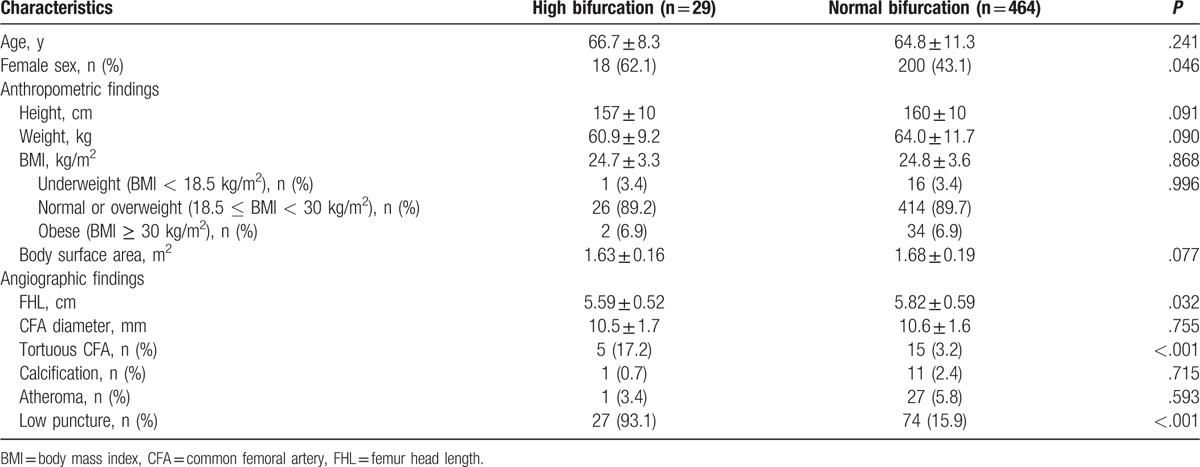
Baseline clinical characteristics between patients with high bifurcation and normal bifurcation.

### 3.2 Clinical and angiographic characteristics of study patients with normal bifurcation

**Figure 3:**
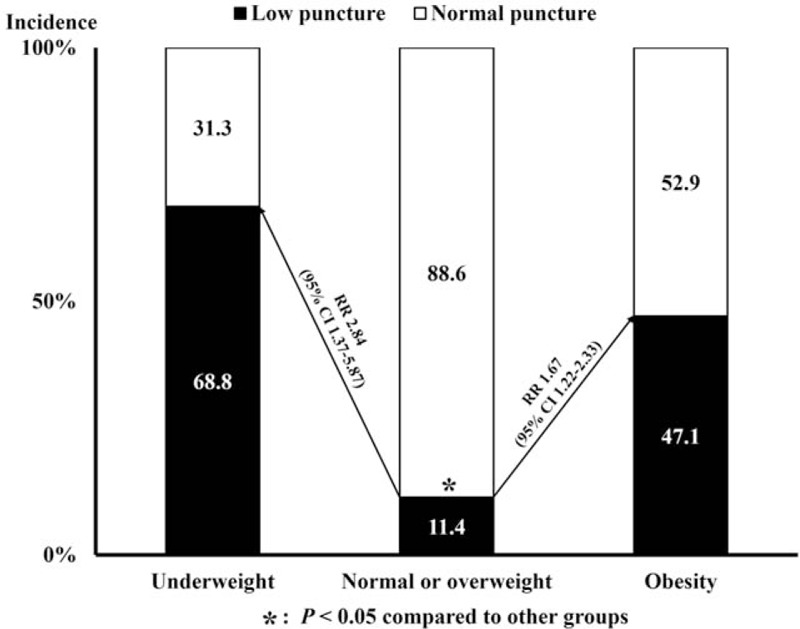
Risk of low puncture according to body mass index. CI = confidence interval, RR = relative risk.

Comparisons of clinical and angiographic findings between the low and proper puncture groups are shown in Table 2. Mean age was similar between the low and proper puncture groups (65.7 ± 11.5 years vs. 64.5 ± 11.3 years, *P* = .419). The proportion of female was significantly higher in the low puncture group compared with the proper puncture group (54.0% vs. 41.0%, *P* = .038). The height was shorter in the low puncture group compared with the proper puncture group (158 ± 9 cm vs. 160 ± 9 cm, *P* = .047), but there were no significant differences in body weight or BMI (*P* > .05 for each). Interestingly, the frequencies of underweight and obesity were significantly higher in the low puncture group compared with the proper puncture group (36.5% vs. 5.9%, *P* < .001). In addition, 68.8% of patients with underweight (RR to normal or overweight, 2.84; 95% CI, 1.37–5.87) and 47.1% of patients with obesity (RR to normal or overweight, 1.67; 95% CI, 1.22–2.33) had low puncture, while 11.4% of patients with normal or overweight did (Fig. 3). There were no significant differences in angiographic findings, such as FHL, CFA diameter, or vascular characteristics (tortuosity, calcification, and atheroma), between the 2 groups (*P* > .05 for each).

**Table 2:**
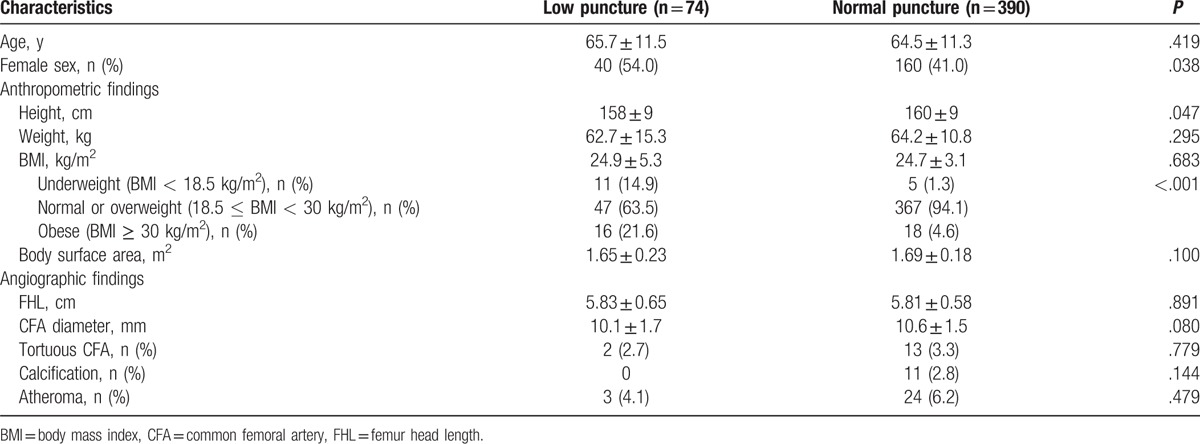
Clinical and angiographic characteristics of study patients having normal bifurcation.

### 3.3 Periprocedural complications of patients with normal bifurcation

Puncture-related complications are shown in Table 3. There were 43 cases of hematoma and 6 cases of bleeding associated with CFA puncture. There were no cases of bleeding requiring transfusion and pseudoaneurysm formation. The incidences of overall procedure-related complications were not significantly different between the 2 groups (43 [11.0%] vs. 6 [8.1%], *P* = .306).

**Table 3:**
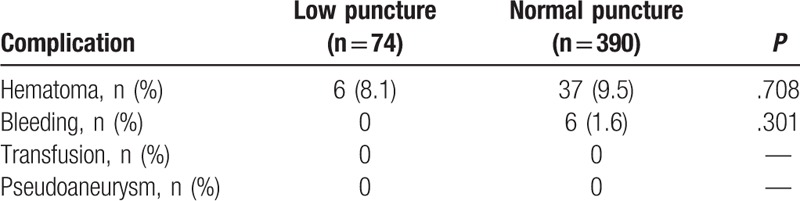
Periprocedural complications in study patients having normal bifurcation.

### 3.4 Independent risk factors for low puncture in patients with normal bifurcation

Multivariable logistic regression analysis showed underweight or obesity was significantly associated with high risk of low puncture even after controlling for clinical covariates (OR, 9.10; 95% CI, 4.77–17.35; *P* < .001). Increased CFA diameter was associated with reduced risk of low puncture (OR, 0.81; 95% CI, 0.68–0.97; *P* = .021). Age, sex, and FHL were insignificant factors for low puncture in multivariable analysis (Table 4).

**Table 4:**
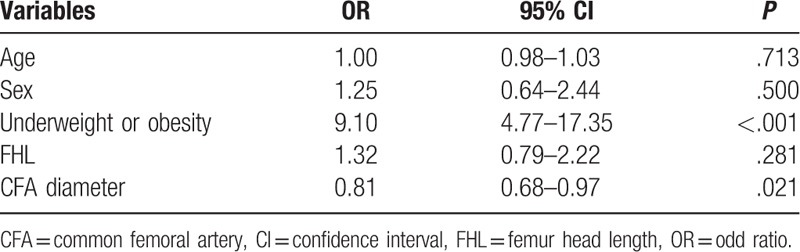
Independent risk factor for low puncture in patients with normal bifurcation.

### 3.5 Clinical and angiographic findings according to BMI in patients with normal bifurcation

Clinical characteristics and femoral angiographic findings of the patients were compared according to BMI (<18.5, 18.5–29.9, and ≥30 kg/m^2^) (Table 5). As BMI increased, the proportion of females (*P* = .030) and CFA diameter (*P* = .023) increased. The average distance from IBFH to the CFA puncture site was longer in patients with normal BMI or overweight than in those with underweight or obesity (*P* = .030). Age, FHL, and CFA diameters were not associated with BMI (*P* > .05 for each). Trigonometric calculation showed that the average distance from IBFH to the CFA puncture site was 0.5 to 2.59 cm shorter in underweight patients compared with those of normal weight or overweight patients (Supplementary Data).

**Table 5:**
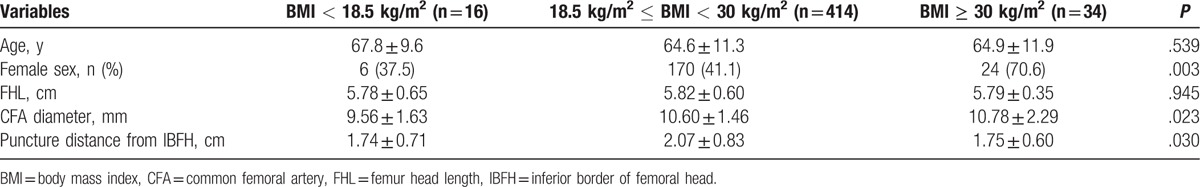
Clinical and angiographic findings according to BMI in patients with normal bifurcation.

## 4 Discussion

Our results showed that underweight or obese patients were at high risk of low puncture, compared with normal or overweight patients during ICA using the femoral artery approach. This association between BMI and the risk of low puncture was independent of covariates, including sex, age, FHL, and femoral artery status (diameter, tortuosity, calcification, and atheroma). All of these important clinical factors were not related to risk of low puncture. Patients with underweight or obesity divided according to BMI showed a shorter distance between the puncture site and IBFH than those with normal BMI or overweight. To our best knowledge, this is the first study showing the direct association between BMI and the risk of low puncture in patients undergoing ICA using femoral artery access.

**Figure 4:**
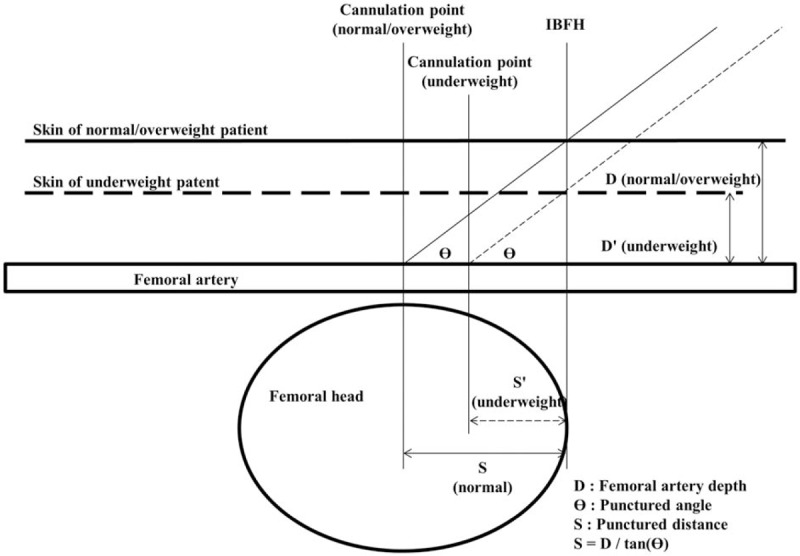
Simplified diagrams of femoral artery puncture. BMI = body mass index.

Based on the fact that low puncture is related to higher incidence of vascular complications, our findings were in line with those of a prior study showing that PCI access-related vascular complications were highest in extremely thin and morbidly obese patients.^[13]^ Simple geometric calculation can explain the high risk of low puncture in underweight patients. As femoral artery depth is positively correlated with BMI,^[18]^ it is shallower in underweight patients than in normal patients. Thus, underweight patients are at high risk of low puncture with the conventional puncture technique according to trigonometric calculation (Fig. 4). Thus, puncture sites should be slightly proximal to IBFH in underweight patients to avoid low puncture. From our calculation (Supplementary Data), as the difference in average distance from IBFH to the CFA puncture site was 0.5 to 2.59 cm (mean = 1.32 cm), about 1 finger width separation from the IBFH may be appropriate for proper puncture. However, this geometric consumption may not be directly applied in obese patients, because femoral artery depth and puncture angle can be easily interrupted during the procedure by a large amount of jiggling fat in the groins. Also, this unreliability of femoral artery depth and puncture angle can interrupt the conventional puncture technique. Therefore, transradial access or the use of the ultrasonography-guided femoral artery puncture technique may be preferred in morbidly obese patients for more reliable and safe puncture.^[19,20]^

Another effort to avoid low puncture was to find predictors of high femoral artery bifurcation. In our study, 29 patients (5.8%) had high bifurcation, which may have increased the risk of low puncture. Indeed, low puncture occurred in most of these patients (93.1%). Our results showed that women with a short FHL and a tortuous iliofemoral artery are more likely to have high bifurcation of the femoral artery. More careful approaches should be considered in these patients.

Even though radial artery puncture is a well-known safer technique,^[21]^ it cannot be totally replaced with femoral artery puncture in ICA. Compared with radial access, femoral access has several strengths.^[6,22]^ First, life-saving devices in critically ill patients, such as intra-aortic balloon pump placement or extracorporeal membrane oxygenation, are only available via the femoral artery. Second, procedural time is shorter with less radiation exposure in the femoral approach. Third, bearing capacity of a catheter is stronger in the femoral artery approach with easier manipulation of a catheter. In addition, safe femoral artery puncture is more important in critically ill patients. Because such patients are at high risk of bleeding or disseminated intravascular coagulation,^[23,24]^ failed femoral artery puncture can be life-threatening to them. As ultrasound-guided femoral artery puncture is not frequently available, fluoroscopy-guided femoral artery puncture is still efficient even in critically ill patients.^[25,26]^ Therefore, our results deserve clinical attention, and can be a great help in cardiac intervention.

### 4.1 Study limitations

Our study has several limitations including its retrospective design. First, this study was performed at a single center, and a single cardiologist performed femoral puncture. Therefore, factors, such as race and socioeconomic status affecting obesity and physician's ability, could affect procedural outcomes. Second, study population was relatively small, and statistical power was limited. By this reason, there might be a possibility that the incidence of puncture site complications did not show significant differences between normal and low puncture groups. Lastly, the measurements from femoral angiography may differ from actual values. Radiation angle, overlapping between adjacent structures, and shades of dye could affect the measurements.

## 5 Conclusion

In patients with normal CFA bifurcation, underweight or obesity was associated with increased risk of low puncture using the fluoroscopy-guided femoral artery approach. The puncture site should be chosen about 1 finger width more proximal to IBFH for ICA in such patients.

## Supplementary Material

Supplemental Digital Content
