# Supplementary material for: Body mass index and the risk of low femoral artery puncture in coronary angiography under fluoroscopy guidance
Source: Medicine (Baltimore). 2018 Mar 2;97(9):e0070. doi: 10.1097/MD.0000000000010070 (PMC5851760; doi:10.1097/MD.0000000000010070)
Supplement: Supplemental Digital Content [file medi-97-e0070-s001.doc]

Supplementary Data. Trigonometric calculation of puncture distance (cm) according to femoral artery depth and puncture angle

| **Puncture angle** | **Femoral artery depth** | | | |
| --- | --- | --- | --- | --- |
|  | **Underweight**  **(BMI < 18.5 kg/m^2^)** | | **Normal/overweight**  **(18.5 ≤ BMI < 30 kg/m^2^)** | |
|  | **0.5 cm** | **1 cm** | **1.5 cm** | **2 cm** |
| **30°** | 0.87 | 1.73 | 2.60 | 3.46 |
| **31°** | 0.83 | 1.66 | 2.50 | 3.33 |
| **32°** | 0.80 | 1.60 | 2.40 | 3.20 |
| **33°** | 0.77 | 1.54 | 2.31 | 3.08 |
| **34°** | 0.74 | 1.48 | 2.22 | 2.97 |
| **35°** | 0.71 | 1.43 | 2.14 | 2.86 |
| **36°** | 0.69 | 1.38 | 2.06 | 2.75 |
| **37°** | 0.66 | 1.33 | 1.99 | 2.65 |
| **38°** | 0.64 | 1.28 | 1.92 | 2.56 |
| **39°** | 0.62 | 1.23 | 1.85 | 2.47 |
| **40°** | 0.60 | 1.19 | 1.79 | 2.38 |
| **41°** | 0.58 | 1.15 | 1.73 | 2.30 |
| **42°** | 0.56 | 1.11 | 1.67 | 2.22 |
| **43°** | 0.54 | 1.07 | 1.61 | 2.14 |
| **44°** | 0.52 | 1.04 | 1.55 | 2.07 |
| **45°** | 0.50 | 1.00 | 1.50 | 2.00 |

Gray zone indicates ideal puncture distance under conventional femoral puncture technique. Considering femoral artery depth as 1~1.5 cm and puncture angle as 30~45° in normal or overweight subjects (18.5 ≤ BMI < 30 kg/m^2^), calculated puncture distance (distance between artery cannulation site and IBFH) is ranged as 1.5~3.46 cm (= ideal puncture site). In underweight patients (BMI < 18.5 kg/m^2^), femoral artery depth is estimated as 0.5~1 cm, and the puncture distance can be calculated as 0.5~1.73 cm in the same puncture angle. Thus, the difference in puncture distance between normal/overweight and underweight subjects is ranged as 0.5~2.59 cm (mean = 1.32 cm = about 1 finger width). From this calculation, about 1 finger width proximal to IBFH is recommended to locate the needle tip at the ideal puncture site of femoral artery in underweight patients.
